# P-1914. Clinic-Based HIV Learning: The Effectiveness of a Mini-Lecture Series

**DOI:** 10.1093/ofid/ofaf695.2083

**Published:** 2026-01-11

**Authors:** Jacqueline E Sherbuk, Ambika Eranki

**Affiliations:** University of South Florida, Tampa, FL; Morani College of Medicine, University of South Florida, Tampa, Florida

## Abstract

**Background:**

HIV primary care is an essential skill for infectious disease physicians. Trainees gain clinical experience through longitudinal outpatient HIV care, a requirement for ACGME-accredited Infectious Diseases (ID) fellowship programs. However, incorporating didactic education during busy clinic sessions can be a challenge to providing consistent, thorough HIV education.

**Figure 1.**
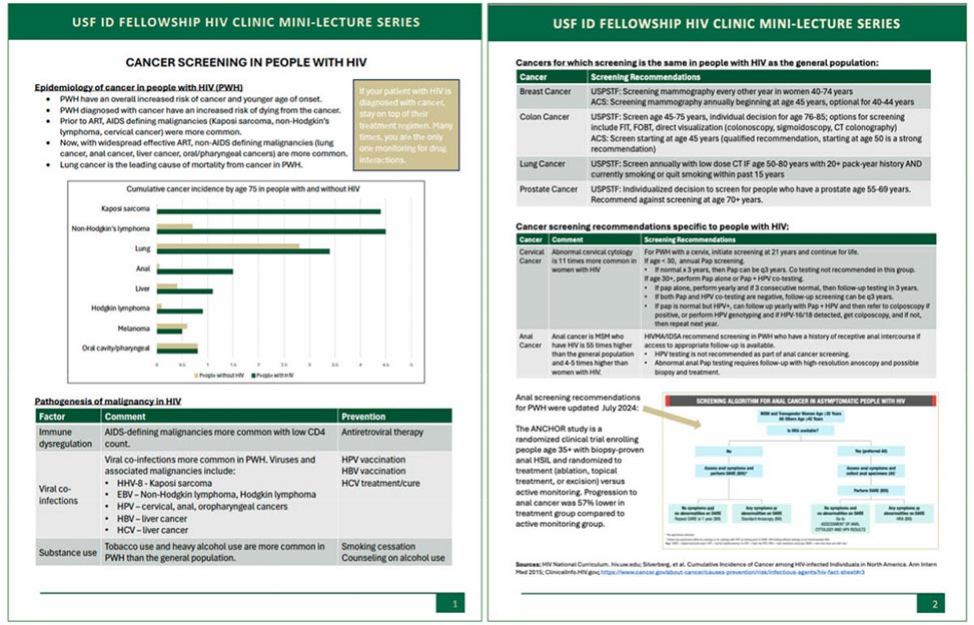
Example handout an HIV mini-lecture on the topic of cancer screening in PWH

**Figure 2.**
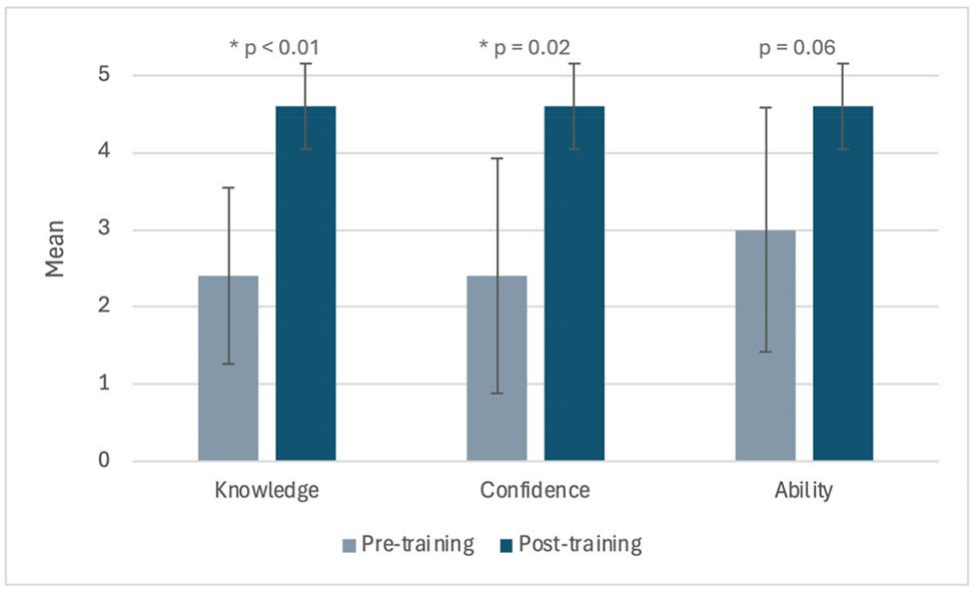
Change in knowledge, skills, and attitudes related to care of PWH pre and post intervention, as rated by ID fellow self-report. Participants were asked to rate “knowledge of HIV”, “confidence in managing PWH” and “ability to provide care for PWH” on a scale of 1 (lowest) to 5 (highest) before and after training.

**Methods:**

ID faculty identified key topics in HIV education, using the HIV National Curriculum, Infectious Disease Board Review topics, and In-Training Exam results. For each topic, a handout was created to guide a 10-minute didactic session delivered weekly during HIV clinic. During the year, ID fellow suggestions were incorporated into future topics. Participating ID fellows completed an anonymous evaluation regarding effectiveness, engagement, and relevance. Impact on fellows’ knowledge, skills, and attitudes was ranked on a 1 to 5 (highest) scale and pre and post test scores were compared using T-test. Participants were also invited to share qualitative feedback through comments.

**Table 1.**
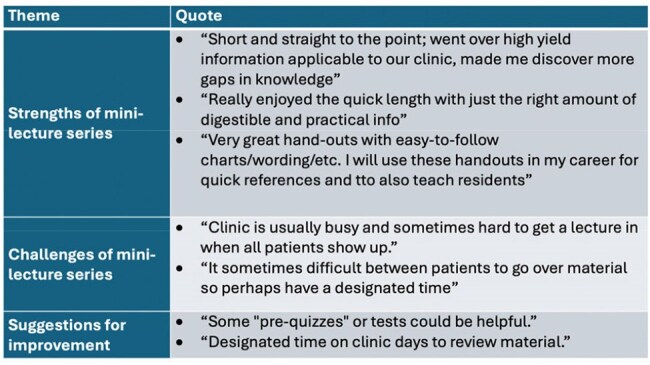
Select quotes from ID fellow evaluationa of the mini-lecture series.

**Results:**

From July 2024 to April 2025, 27 HIV mini-lectures were delivered [Figure 1]. Six ID fellows completed the evaluation, out of seven fellows who participated (85.7%). Participants found the mini-lecture series to be effective, engaging, organized, appropriate to level of training, and excellent in quality. Participants felt more knowledgeable, confident, and able to manage patients with HIV after participating in the mini-lecture series [Figure 2]. Participants appreciated the length, high-yield information, and handouts, though despite the brevity of lectures, fellows still found mini-lectures difficult to fit into the clinic workflow at times [Table 1].

**Conclusion:**

The HIV mini-lecture series improved ID fellows’ knowledge, confidence and ability to care for people with HIV. The format of brief lectures was acceptable to fellows, though requires ongoing evaluation of optimal time to incorporate material. This mini-lecture series could be adopted by other HIV medical education training programs.

**Disclosures:**

All Authors: No reported disclosures

